# RNF207 promotes colorectal cancer growth by regulating the Hippo–YAP pathway via enhanced MST1 ubiquitination and degradation

**DOI:** 10.1007/s00535-026-02440-2

**Published:** 2026-05-27

**Authors:** Meng Wang, Xing Wen, Mei Yang, Jie Zhang, Jianhui Gu, Tianqi Lu

**Affiliations:** 1 Center of Gastrointestinal and Minimally Invasive Surgery, Department of General Surgery, The Third People’s Hospital of Chengdu, Chengdu, 610031 China; 2 Department of Gastroenterology, The Third People’s Hospital of Chengdu, Chengdu, 610014 China; 3 Obesity and Metabolism Medicine-Engineering Integration Laboratory, Department of General Surgery, The Third People’s Hospital of Chengdu, Chengdu, 610031 China; 4 Center of Gastrointestinal and Minimally Invasive Surgery, Department of General Surgery, Affiliated Hospital of Southwest Jiaotong University, The Third People’s Hospital of Chengdu, Chengdu, 610031 China

**Keywords:** Colorectal cancer, RNF207, Hippo–YAP, MST1, Ubiquitination

## Abstract

**Background:**

Colorectal cancer ranks second in global mortality rates, yet its molecular pathogenesis remains incompletely understood. Accumulating evidence indicates that E3 ubiquitin ligases modulate the activity and stability of critical oncoproteins via ubiquitination, positioning these enzymes as promising therapeutic targets for cancer intervention. The E3 ligase Ring finger protein 207 (RNF207), characterized as a regulator involved in heart disease, has not been investigated in colorectal cancer.

**Methods:**

We constructed RNF207 overexpression and knockdown colorectal cancer cell lines, and investigated the effects of RNF207 on the proliferation and migration of colorectal cancer cells through experiments such as CCK8, colony formation, migration, and invasion assays. In colorectal cancer cell lines and human colorectal cancer tissues, the influence of RNF207 on the Hippo–YAP signaling pathway was extensively investigated. The mechanism by which RNF207 regulates P-YAP and MST1 was explored through Co-IP and ubiquitination experiments. Finally, the effect of RNF207 on the growth of colorectal cancer was investigated in nude mice.

**Results:**

Here, we demonstrate that elevated RNF207 expression in colorectal tumors correlates with poor prognosis. Functional studies revealed that RNF207 enhances colorectal cancer cell proliferation, migration. Mechanistically, we identified that RNF207 promotes colorectal cancer progression through MST1-dependent regulation of Hippo–YAP signaling. Specifically, RNF207 interacts with MST1 and induces its proteasomal degradation via K48-linked ubiquitination dependent on the activity of E3 ligase, thereby attenuating YAP phosphorylation and activating YAP-driven transcription. Furthermore, the protein level of RNF207 was significantly negatively correlated with the protein levels of MST1 and P-YAP in colorectal tumors, and reducing the expression of RNF207 can effectively inhibit the growth of colorectal cancer.

**Conclusions:**

Collectively, our findings uncover a novel oncogenic function of RNF207 in colorectal cancer, whereby it facilitates MST1 degradation through K48 ubiquitination, leading to YAP hyper-activation. Targeting RNF207–MST1–YAP axis may represent a potential therapeutic for colorectal cancer treatment.

**Supplementary Information:**

The online version contains supplementary material available at https://doi.org/10.1007/s00535-026-02440-2.

## Introduction

Colorectal cancer (CRC) ranks as the third most prevalent malignancy globally and is the second leading cause of cancer-related mortality [1]. The incidence rates for CRC in the United States from 2016 to 2020 were 35.3 per 100,000 people [2]. The prevalence of CRC is demonstrating a tendency toward a younger age of onset, with the number of patients under 50 years old rising steadily year after year [3]. CRC occurs mainly in the mucosal epithelium of the colon and rectum and exhibits considerable heterogeneity [4]. Multiple factors, such as genetics alterations, the environment, and cell metabolic reprogramming, play pivotal roles in CRC pathogenesis [5, 6]. Although current treatment options including surgery, chemotherapy, radiotherapy, molecular-targeted therapy, and immunotherapy decrease the risk of CRC-related mortality to some extent, elucidating the genetic, epigenetic, and metabolic landscapes of CRC continues to be indispensable [7].

The Hippo pathway is critical for tumorigenesis initiation and frequently disrupted in CRC due to genetic mutations and altered expression of its core components, making targeting its upstream regulators or core elements a potential innovative strategy to modulate signaling dysfunction in CRC [8]. In mammals, the Hippo pathway is composed of several key proteins, including mammalian STE20-like protein kinase (MST1/2), large tumor suppressor 1/2 (LATS1/2), and their respective adaptor proteins salvador homolog 1 (SAV1) and MOB kinase activator 1 (MOB1), as well as two transcriptional coactivators Yes-associated protein (YAP) and transcriptional coactivator with PDZ-binding motif (TAZ), which are primary functional exporters of Hippo signaling. When Hippo signaling is activated, the MST1/2–SAV1 complex phosphorylates and activates LATS1/2–MOB1 complex, which further phosphorylates and inhibits the activity of YAP and TAZ by binding to scaffold protein 14-3-3 in cytoplasm and subsequent degradation by ubiquitin proteasome system. When Hippo pathway is off, unphosphorylated YAP and TAZ translocate to the nucleus to interact with and activate TEADs (TEA domain transcription factors), which further activate genes responsible for cell proliferation, metastasis and malignant transformation [9, 10]. The therapeutic development centering on YAP/TAZ activity has always been a highly active field [11, 12].

RNF207 is a RING-type zinc-finger E3 ligase, composed of RING domain, B-box 1 domain, B-Box C-terminal (BBC) domain, and C-terminal homologous region (CHR) [13]. To date, research focusing on the function of RNF207 has been scarce. The most highlighting function of RNF207 is in the field of cardiac diseases [13–16]. RNF207 is discovered and proved to regulate heart QT interval through eliminating misfolded hERG (human ether-à-go-go related gene), which is critical for ventricular repolarization, and RNF207-hERG module also accounts for action potential duration in cardiac electrophysiology[14]. Moreover, RNF207 exacerbates pathological cardiac hypertrophy via post-translational modification of TAB1, but promotes cellular concentration of adenosine triphosphate (ATP) and mitochondrial dysfunction in cardiomyocytes [15, 16]. Considering cancer research, RNF207 stands out in prognosis prediction of prostate cancer, endometrial cancer and juvenile cervical clear cell adenocarcinoma unrelated to human papillomavirus [17–19]. However, the functional researches of RNF207 in cancer has not been seen, not to mention colorectal cancer.

Here in the present research, we identified RNF207 upregulated in CRC cancerous tissues and its lower expression might benefit the survival of CRC patients. We next performed CCK-8, colony formation, and Transwell assays, and proved RNF207 promoted CRC cell progression in vitro. Mechanistically, RNF207 interacted with MST1 and accelerated its degradation through K48-linked ubiquitination, thereby lowering the phosphorylation of YAP protein and ultimately promoting tumor progression. Furthermore, the protein level of RNF207 was significantly negatively correlated with the protein levels of MST1 and P-YAP in colorectal tumors, and reducing the expression of RNF207 can effectively inhibit the growth of colorectal cancer. Our findings firstly revealed the function and mechanism of action of RNF207 in CRC and deepen the understanding of CRC pathology and provided new therapeutic strategies for CRC treatment.

## Materials and methods

### Bioinformatics analyses

The CRC transcriptome data were downloaded from the TCGA and GEO database (GSE146889) and then transformed into TPM using R package easyTCGA (Version 0.1.0). The expression of RNF207 in colorectal cancer was analyzed, and the visualization map was plotted, which was statistically tested by wilcox.test. The overall survival time and disease-free interval were obtained from the TCGA official website. Kaplan–Meier estimation and curve drawing using R package (survival) and (survivminer).

### Human CRC and paracancerous tissue samples

Cancer and paracancerous tissue samples from CRC patients were collected after informed consent of the patients and approval by the Ethics Committee of the Third People’s Hospital of Chengdu.

### Cell culture and treatment

The adopted CRC cell lines, HCT116 (RRID: CVCL_0291) and DLD-1 (RRID: CVCL_0248), were obtained from BeNa Culture. The two CRC cells were cultured in RPMI-1640 medium (PM150110, Procell, China) and HEK293T cells were cultured in Dulbecco’s modified eagle medium (DMEM; PM150210, Procell, China), with 10% fetal bovine serum (FBS; FBS-S500, NEWZERUM, China) and 1% penicillin–streptomycin solution (PB180120, Procell, China) inside. The incubation condition was set as 37 ℃ with 5% CO_2_ atmosphere. CHX (cyclohexane; HY-12320, MCE, USA), MG132 (HY-13259, MCE, USA), CQ (chloroquine; HY-17589A, MCE, USA), and PY-60 (HY-141644, MCE, USA) were supplemented in the culture medium with the working concentration of 50 μM, 25 μM, 50 μM, and 10 μM.

### Plasmid construction and lentivirus infection

All plasmids used in this study were purchased from companies such as PAIVIBIO or Miaoling Plasmid. To construct cell lines consistently expressing target sequences, HEK293T cells were inoculated, cultured, and transfected with mixed expression plasmids with two packaging plasmids, pMD2.G and psPAX2, and polyethyleneimine (PEI, 23966-1, Polysciences, USA) served as transfection reagents. 48 h after transfection, the supernatant of culture medium was collected and filtered with 0.22 μm filter membrane (SLGPR33RB, Millipore, USA). Subsequently, well-cultured HCT116 or DLD-1 cells at 30 ~ 50% confluency were infected with lentiviral particles in filtered medium in the presence of 12 μg/mL polybrene (HB-PB-500, Hanbio, China) for 6 h. When the infected cells were subcultured, 5 μg/mL puromycin (BLGE320-25MG, BioLight, China) was supplemented for 48 h to select positive cells.

### Dual-luciferase reporter assay

For dual‑luciferase reporter assays, the wild‑type or mutant RNF207 promoter sequences were cloned into the pGL3‑Basic vector. HEK293T cells were co‑transfected with reporter construct, pSV40-Renilla plasmid, and FOXM1 overexpression or control vector using PEI. After 48 h, cells were lysed, and firefly and Renilla luciferase activities were measured sequentially using the Dual‑Luciferase Reporter Assay System (BRK0044, Abclonal). Relative luciferase activity was calculated as the ratio of firefly to Renilla luminescence.

### Real-time quantitative polymerase chain reaction (qPCR)

To extract RNA from cells or tissues, TRIzol reagent (4,992,730, Tian Gen, China) was adopted and ultra-sonication facilitated the fragmentation of tissues. Following the manufacturer’s guidelines, the total RNA was extracted and further reversely transcribed using HiScript II 1st Strand cDNA Synthesis Kit (R212-01, Vazyme, China). Diluted cDNA was mixed with SYBR Green PCR Master Mix (Q711-02, Vazyme, China) and the primers, and further subjected to LightCycler 480 System (Lepu Medical, China) as per the established protocol. The primers used in this study were recorded in Supplementary Table S1.

### Protein extraction and Western blotting

The protein from cells and tissues was respectively extracted by SDS lysis buffer and RIPA buffer facilitated with ultra-sonication, and further subjected to quantification by BCA Protein Assay Kit (23225, Thermo Fisher Scientific, USA). For subcellular fractionation, CRC cells were harvested, washed with ice-cold PBS, and resuspended in hypotonic buffer (10 mM HEPES pH 7.9, 1.5 mM MgCl_2_, 10 mM KCl, 0.5 mM DTT, and protease inhibitors). After 10 min incubation on ice, 0.1% NP-40 was added, and the lysate was vortexed for 10 s, then centrifuged at 12,000 g for 1 min at 4 ℃. The supernatant was collected as the cytoplasmic fraction. The nuclear pellet was washed once with hypotonic buffer, resuspended in high-salt buffer (20 mM HEPES pH 7.9, 400 mM NaCl, 1.5 mM MgCl_2,_ 0.2 mM EDTA, 25% glycerol, 0.5 mM DTT, and protease inhibitors), incubated on ice for 30 min with intermittent vortexing, and centrifuged at 16,000 g for 15 min at 4 ℃. The supernatant was collected as the nuclear fraction. Purity of the fractions was verified by Western blotting using GAPDH (cytoplasmic marker) and Lamin B (nuclear marker). The protein separation was achieved in SDS-PAGE gels and further transferred to PVDF membranes (IPVH00010, Millipore, USA). The membranes were subsequently blocked in skimmed milk and washed by 1 × TBST buffer. The first antibodies were incubated with the membrane at 4 ℃ overnight and the second antibodies (115-035-003 or 111-035-003, Jackson ImmunoResearch Laboratories, USA) were correspondingly incubated at room temperature for 1 h. Finally, the visualization was proceeded in the ChemiDoc^™^ XRS + Imaging System (Bio-Rad, USA) with ECL Western blotting Substrate kit (SQ201, Epizyme Biotech, China). The antibodies used in this study are recorded in Supplementary Table S1.

### Cell counting kit 8 (CCK-8) assays

CCK-8 reagents (C0039, Beyotime, China) were adopted to assess the cell proliferation rate and the manufacturer’s protocol was followed. The cells were inoculated into 96-well plates at a density of 5000 HCT116 or DLD-1 cells per well, and every three wells were set as duplicates. The test time point was set at 0, 1, 2, 3, and 4 days after inoculation. When doing the test, every well was added CCK-8 solution equally to 10% of total medium volume and subsequently put into 37 ℃ incubator for 2 h. Optical density (OD) value was obtained at the wavelength of 450 nm and used for further investigation.

### Colony formation

2000 HCT116 or DLD-1 cells were inoculated into 6-well plates and cultured at 37 ℃ for 10 days. During the period, the culture medium was replaced every three days to guarantee optical growth. Next, the plates were washed and fixed by 4% paraformaldehyde (G1101-500ML, Servicebio, China) for 20 min, followed by staining in 0.1% crystal violet staining solution (CAS No. 548-62-9, Sinopharm, China) for 15 min. After sufficient washing, Nikon D7000 camera (Japan) was adopted for taking picture.

### Transwell assay

6 × 10^5^ HCT116 or 1.2 × 10^5^ DLD-1 cells were first collected and resuspended in serum-free medium, and inoculated into the upper chambers of Corning Transwell permeable supports (REF 3421, Corning, USA), where membranes had 8.0 μm pore size. Then, 600 μL of complete medium was added into the lower chamber and the whole chambers were put into 37 ℃ incubator with 5% CO_2_ for 24 h. The membrane was washed, fixed in 4% paraformaldehyde, and stained with 0.1% crystal violet solution. After removing the cells from upper membrane, the cells migrating to the lower membrane were pictured by microscope (Mshot, MF52-N + MDX10). For Transwell invasion assay, 5 μg Matrigel Basement Membrane Matrix (G4130-5ML, Servicebio, China) was first added to the upper membrane to simulate an extracellular matrix before inoculation. The subsequent procedures were the same as above description. The cell number on the lower membrane was counted and further analyzed.

### Subcutaneous tumor-bearing mouse models

Mice were housed in a specific pathogen-free, temperature-regulated environment with room temperature of 22–24 ℃, humidity of 40–70%, and alternating lighting time for 12 h, and they were free to drink and eat. 5*10^6^ HCT116 cells suspended in 200 μL PBS were inoculated into BALB/c nude mice aged 4–5 weeks. Tumor volumes were calculated by the formula (0.52 × long diameter × short diameter × short diameter), which were measured by a Vernier caliper at set time point. At the end point (The largest tumor tissue does not exceed 1500 mm^3^), the mice were anesthetized with bromoethanol and the tumors were rapidly isolated.

### Immunohistochemical staining

The paraffin sections were deparaffinized, rehydrated, and processed for antigen retrieval in EDTA buffer or Citrate repair solution under high temperature for 20 min. 10% bovine serum albumin (BSA) was used for blocking at 37 ℃ for 30 min, and subsequent incubation was with antibody (PCNA: Abclonal, A12427; KI67: Servicebio, GB111499-50; RNF207: Sigma-Aldrich HPA028378; p-YAP: Huabio, ET1611-69; MST1:Abclonal, A21842) for overnight at 4 ℃ and secondary antibody for 30 min at 37 ℃. The color was visualized by using 3,3'-Diaminobenzidine (DAB) (Servicebio, G1012) and counter stained by hematoxylin (G1004, Servicebio, China). The images were obtained by light microscope (ML31, MshOT).

### Immunofluorescence staining

CRC cells in plates were fixed with 4% formaldehyde at room temperature for 15 min, washed, permeabilized with 0.1% Triton-X 100 (GC204003, Servicebio, China) in PBS for 20 min and blocked with 8% goat serum in PBS for 30 min. Next, the cells were incubated with antibody at 4 ℃ for overnight. After washing, the corresponding anti-mouse Alexa Fluor 488 secondary antibody (1: 200 dilution, A32723, Invitrogen, USA) and anti-rabbit Alexa Fluor 568 secondary antibody (1: 200 dilution, A11036, Invitrogen, USA) was added and incubated in the dark at 37 ℃ for 1 h. 4′,6-diamidino-2-phenylindole (DAPI; G1012-100ML, Servicebio, China) was counterstained for nuclear visualization. Images were obtained via microscope (Mshot, MF52-N + MDX10).

### Immunoprecipitation (IP) assays

For co-immunoprecipitation (Co-IP) in HEK293T cells, the cells transfected with RNF207 and MST1 overexpression vectors were collected and subjected to IP assay same with the above description. The denatured beads and linked proteins were centrifuged and the supernatant was subjected to Western blotting analysis. For IP assay in HCT116 cells, RNF207 overexpressing and control HCT116 cells were, respectively, collected, subjected to IP assay and Western blotting.

### GST-pulldown assay

HEK293T cells were infected with RNF207 and MST1 expression vectors and lysed in precooled GST lysis buffer with protease inhibitors. The lysates of bait (expressing GST tag) were incubated with GST beads at 4 ℃ for 3 h and then the supernatants of preys were incubated with conjugates of GST beads and baits at 4 ℃ overnight. After washing, the GST beads were denatured at 95 ℃ for 10 min in loading buffer and the supernatant was subjected to Western blotting.

### Ubiquitination assays

293T cells were co-transfected with RNF207 and MST1 overexpression plasmids carrying different tags, as well as ubiquitin molecules carrying a myc tag or ubiquitin molecules of different ubiquitination types. The transfected cells were harvested 24 h post-transfection and the cell pellets were resuspended in 80 μl of IP lysis buffer (20 mM Tris–HCl, pH 7.4; 150 mM NaCl; 1 mM EDTA; 1% NP-40). Subsequently, 10 μl of 10% SDS was added and the mixture was immediately subjected to denaturation at 95 °C for 10–15 min. Following denaturation, the volume was adjusted with 900 μl of IP lysis buffer, followed by sonication to disrupt cellular structures. The lysate was clarified by centrifugation at 4 °C and the supernatant was collected. For immunoprecipitation, the supernatant was incubated with an antibody specific to the MST1 epitope tag for 3–4 h or overnight at 4 °C. The immunoprecipitated complexes were washed three times with a high-salt buffer (20 mM Tris–HCl, pH 7.4; 500 mM NaCl; 1 mM EDTA; 1% NP-40) to remove non-specifically bound proteins. The bound proteins were eluted and they were resolved by SDS-PAGE, followed by Western Blotting (WB) analysis to detect and characterize the protein interactions and modifications.

### Statistical analysis

The data in this research are given as mean ± standard deviation (SD). The significant differences between two groups were assessed by the Student’s t-test, in which a two-sided *P* value of less than 0.05 was applied to indicate the significance. One-way analysis of variance (ANOVA) was utilized for comparisons involving more than two groups.

## Results

### *RNF207* was upregulated in CRC

**Fig. 1:**
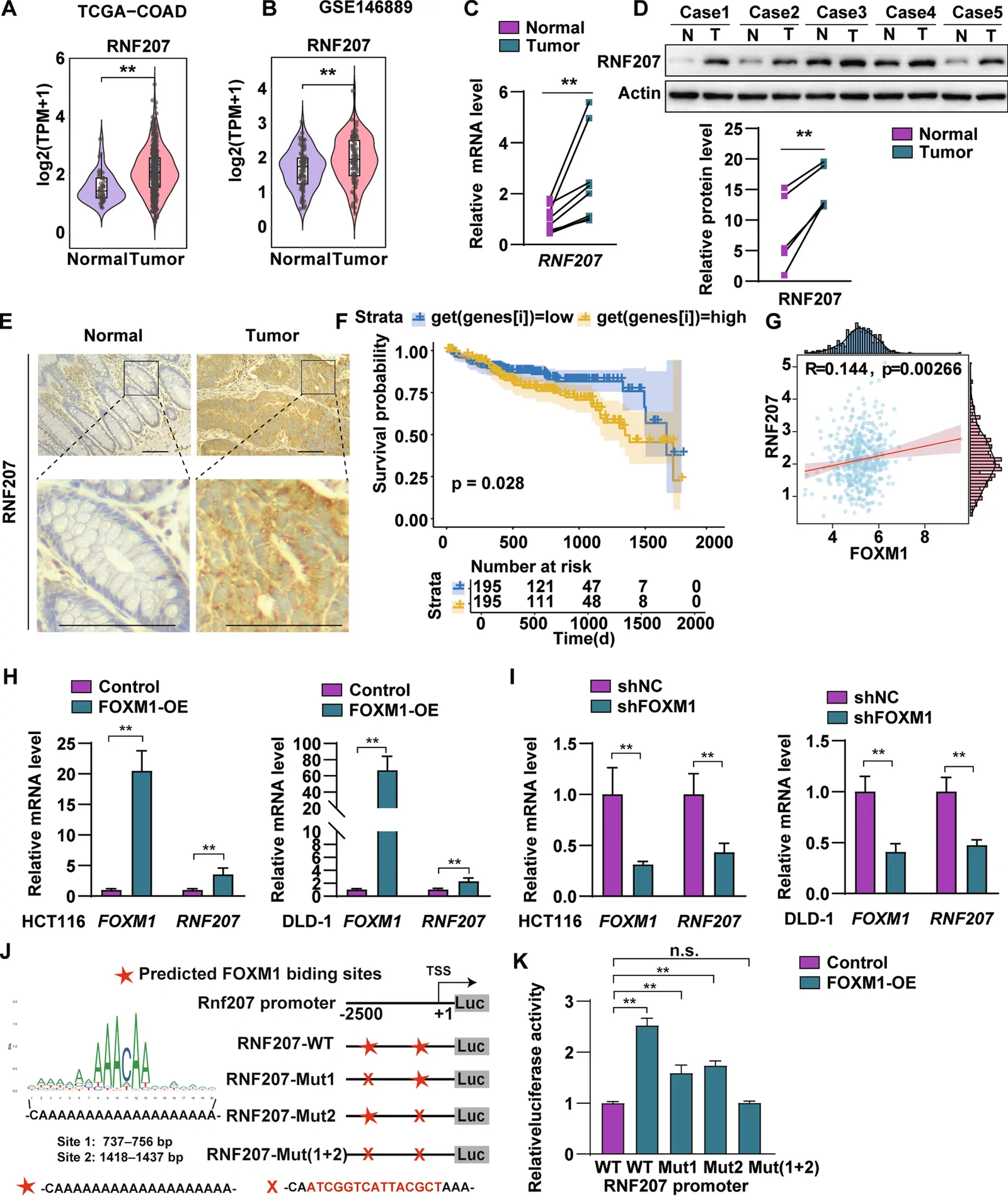
*RNF207* is upregulated in CRC. **A–B** The expression level of *RNF207* in CRC from TCGA database (**A**) and GSE146889 (**B**). **C–D** The mRNA (**C**) and protein expression level (**D**) of RNF207 in normal control and colon tumor tissues from different cases of patients. Actin served as a loading control. **E** Representative immunohistochemical images of RNF207 in cancer tissues and adjacent tissues of colorectal cancer patients. Bar, 50 μm. **F** Kaplan–Meier curves of RNF207 in CRC for progression-free survival. **G** Correlation analysis between FOXM1 and RNF207 mRNA expression levels in TCGA colorectal cancer dataset. **H** Relative RNF207 mRNA levels in HCT116 and DLD-1 cells transfected with FOXM1 overexpression plasmid (FOXM1-OE) or control vector. **I** Relative RNF207 mRNA levels in HCT116 and DLD-1 cells transfected with FOXM1-specific shRNA (shFOXM1) or control shRNA (shNC). **J** Schematic representation of the RNF207 promoter region showing two putative FOXM1 binding sites (Site 1 and Site 2). Luciferase reporter constructs containing the wild-type (WT) promoter or mutants (Mut1, Mut2, Mut1 + 2) are illustrated. **K** Luciferase reporter activity in HEK293T cells co-transfected with the indicated RNF207 promoter constructs and FOXM1 overexpression or control vector. For **C**, **D**, **H** and **I**, two-tailed unpaired Student’s t-test was used. For **K**, one-way ANOVA test was used. **P < 0.01; n.s., not significant

To analyze the correlation of RNF207 with CRC, we performed transcriptional analysis basing on the public database, and found the mRNA expression level of RNF207 was significantly increased in TCGA and GSE146889 database (Fig. 1A, B). We then examined the expression of RNF207 in several paired normal and cancerous tissues of CRC patients and confirmed that RNF207 was higher expressed in cancerous tissues compared with normal tissues (Fig. 1C–E). Moreover, the Kaplan–Meier curves also showed patients with lower RNF207 expression had a better prognosis (Fig. 1F). All these indicated RNF207 might be involved in the progression of CRC and its lower expression might benefit patients’ survival.

To further unravel the molecular mechanism underlying the upregulation of RNF207 in CRC, we performed transcription factor binding site prediction using the JASPAR database. Our analysis specifically focused on FOXM1, a well-recognized oncogenic transcription factor frequently dysregulated in CRC [20]. Moreover, in TCGA COAD dataset, RNF207 expression was positively correlated with FOXM1, which has been proved upregulated in CRC (Fig. 1G). Subsequently, we established CRC cell lines stably overexpressing or silencing FOXM1, and assessed the expression levels of RNF207 in these cells. Quantitative real-time PCR (qRT-PCR) assays demonstrated that FOXM1 overexpression significantly induced RNF207 mRNA levels, whereas FOXM1 knockdown resulted in a marked decrease in RNF207 expression (Fig. 1H, I). To validate the direct transcriptional regulation, we constructed luciferase reporter vectors containing either the wild-type (WT) RNF207 promoter or a promoter with mutated FOXM1 binding motifs (Fig. 1J). Dual-luciferase reporter assays revealed that FOXM1 overexpression robustly enhanced the transcriptional activity of the WT RNF207 promoter. In contrast, mutation of the FOXM1 core binding motifs abrogated this stimulatory effect (Fig. 1K). Collectively, our findings demonstrate that FOXM1 plays a critical role in the transcriptional activation of RNF207 in colorectal cancer.

### Overexpression of RNF207 promotes the proliferation and migration of colorectal cancer cells in vitro

**Fig. 2:**
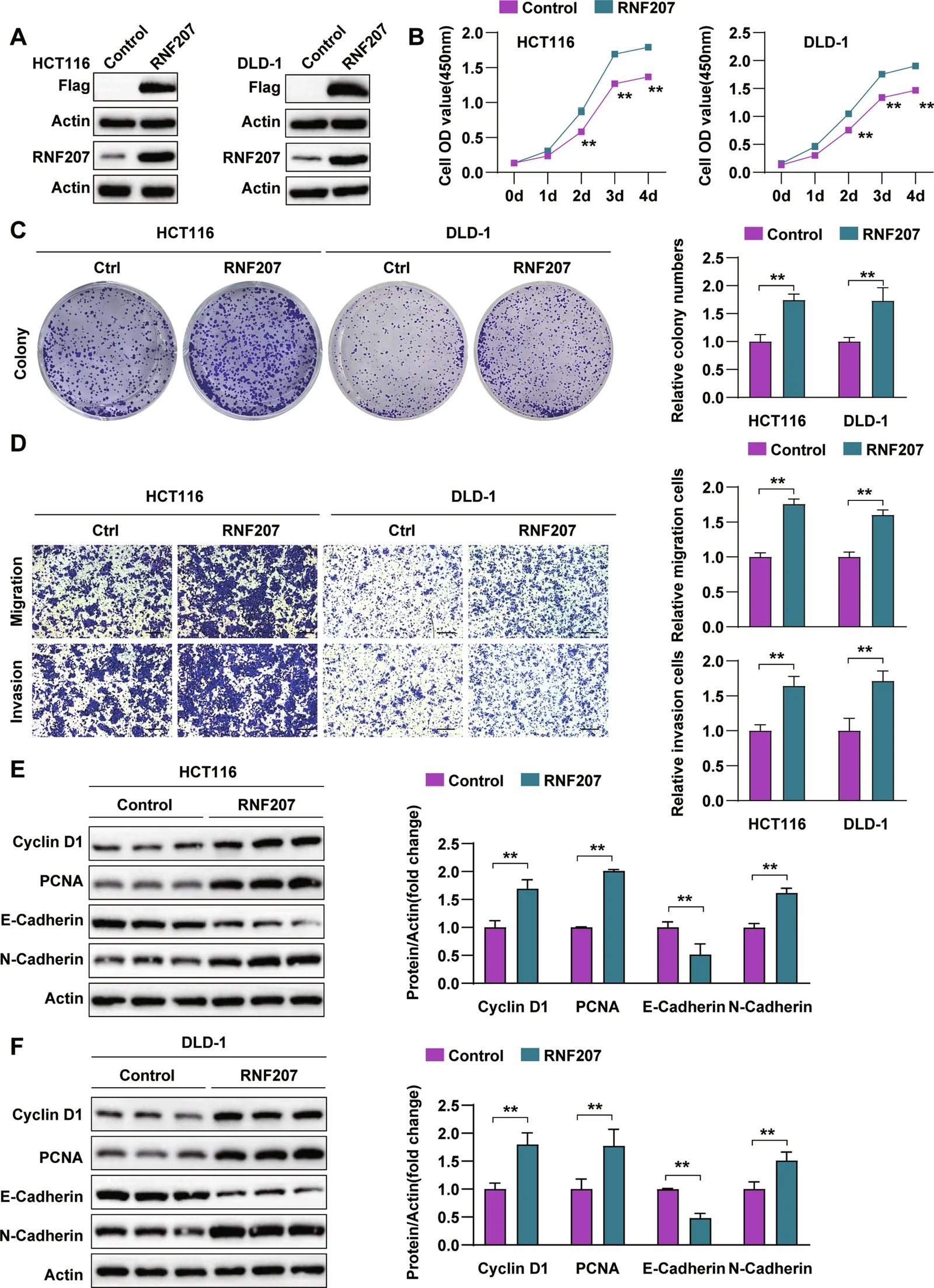
RNF207 overexpression promoted proliferation and migration in CRC cells. **A** Western blotting analysis of RNF207 expression in HCT116 and DLD-1 cells. Actin served as a loading control. **B** CCK-8 assay of RNF207 overexpression in HCT116 (left) and DLD-1 (right) cells. **C** Representative images of colony formation assay (left) and quantification results (right) in RNF207 overexpression HCT116 and DLD-1 cells. **D** Representative images of migration and invasion (left) and quantification results (right) in RNF207 overexpression HCT116 and DLD-1 cells. Bar, 100 μm. **E–F** Western blotting (left) and quantification results (right) of cell proliferation- and metastasis-related proteins in RNF207 overexpression HCT116 (**E**) and DLD-1 (**F**) cells. Actin served as a loading control. For **B–F**, two-tailed unpaired Student’s t-test was used. **P < 0.01

To verify the function of RNF207 in CRC, we constructed RNF207 overexpression cell lines in HCT116 and DLD-1 cells and firstly verified the efficacy of RNF207 overexpression (Fig. 2A). Secondly, we performed CCK-8 and colony formation assays to assess RNF207’s effects on cell proliferation and found that overexpressing RNF207 significantly increased cell growth rate and the settled colony number both in HTC116 and DLD-1 cells, proving RNF207 promoted CRC cell proliferation (Fig. 2B, C). Thirdly, we utilized Transwell assays to value RNF207’s impacts on cell migration and invasion and found that the migrating and invading cell numbers were both multiplied in HCT116 and DLD-1 cells, proving RNF207 also promoted CRC cell metastasis (Fig. 2D). Lastly, Western blotting showed the protein levels of proliferation-promotive PCNA (proliferating cell nuclear antigen) and Cyclin-D1, and metastasis-promotive N-cadherin (neural cadherin) were increased, while the metastasis-suppressive E-cadherin (epithelial cadherin) was decreased due to RNF207 overexpression both in HCT116 and DLD-1 background, these further proved the promotive roles of RNF207 in CRC progression (Fig. 2E, F).

### Knockdown of RNF207 inhibits the proliferation and migration of colorectal cancer cells in vitro

**Fig. 3:**
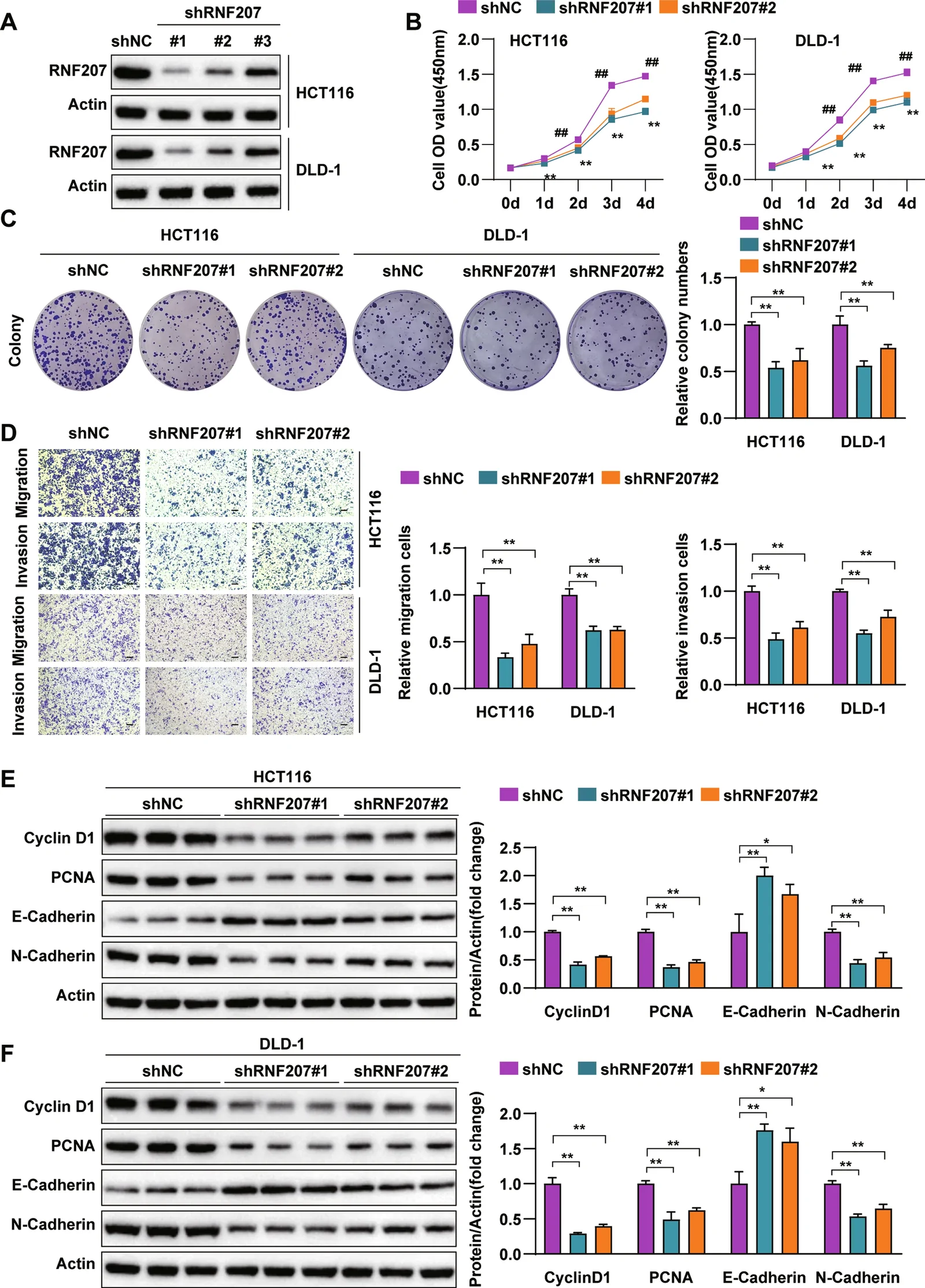
RNF207 knockdown suppressed cell proliferation and migration in CRC cells. **A** Western blotting analysis of RNF207 expression in HCT116 and DLD-1 cells. Actin served as a loading control. **B** CCK-8 assay of RNF207 knockdown (shRNF207#, shRNF207#2) in HCT116 (left) and DLD-1 (right) cells. **C** Representative images of colony formation assay (left) and quantification results in RNF207 knockdown (shRNF207#, shRNF207#2) HCT116 and DLD-1 cells. **D** Representative images of migration and invasion (left) and quantification results (right) in RNF207 knockdown (shRNF207#, shRNF207#2) HCT116 and DLD-1 cells. Bar, 100 μm. **E–F** Western blotting (left) and quantification results (right) of cell proliferation- and metastasis-related proteins in RNF207 knockdown (shRNF207#, shRNF207#2) HCT116 (**E**) and DLD-1 (**F**) CRC cell lines. Actin served as a loading control. For **B-F**, one-way ANOVA test was used. For **B**, *indicates shRNF207#1 vs. Control, ^#^indicates shRNF207#2 vs. Control. ^##^*P* or **P < 0.01; *P < 0.05; n.s., not significant

On the other hand, we also established RNF207 knockdown cell lines in HCT116 and DLD-1 background, Western blotting confirmed that two independent shRNA sequences (sh1 and sh2) achieved efficient knockdown, and these two lines were selected for functional studies (Fig. 3A). Likewise, CCK-8 and colony formation assays showed knocking down RNF207 in CRC cells significantly decreased cell growth rate and the number of colonies (Fig. 3B, C). Transwell assays proved lowering the expressions of RNF207 obviously diminished the migrating and invading cell numbers in CRC cells (Fig. 3D). Besides, the cell proliferation-related PCNA and Cyclin-D1 were decreased, and metastasis-related N-cadherin and E-cadherin were synergistically regulated due to RNF207 knockdown both in HCT116 and DLD-1 cells (Fig. 3E, F). Taking together, we concluded RNF207 exerted oncogenic roles in CRC.

### RNF207 promoted CRC cell progression via activating YAP transcriptional activity

**Fig. 4:**
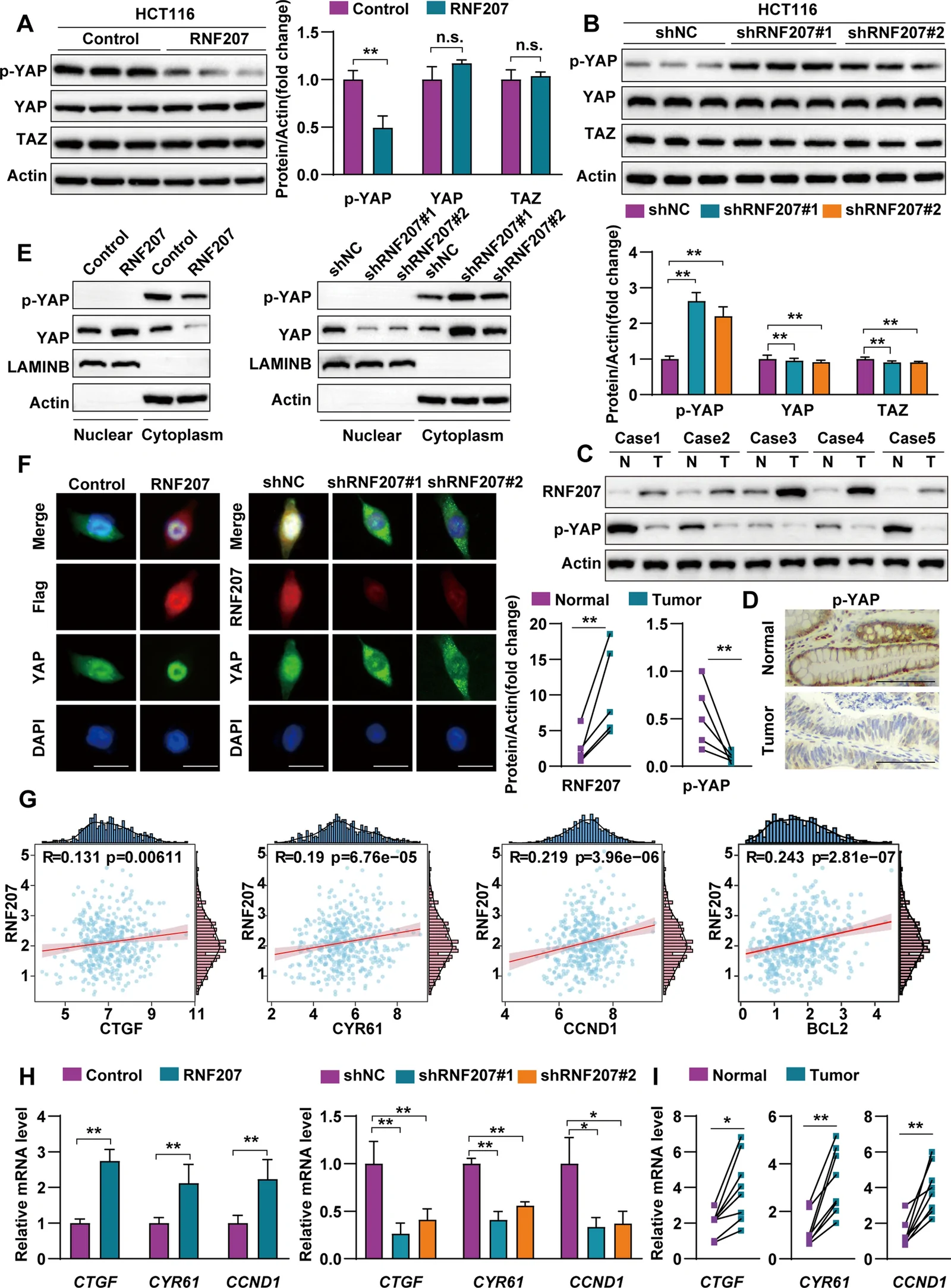

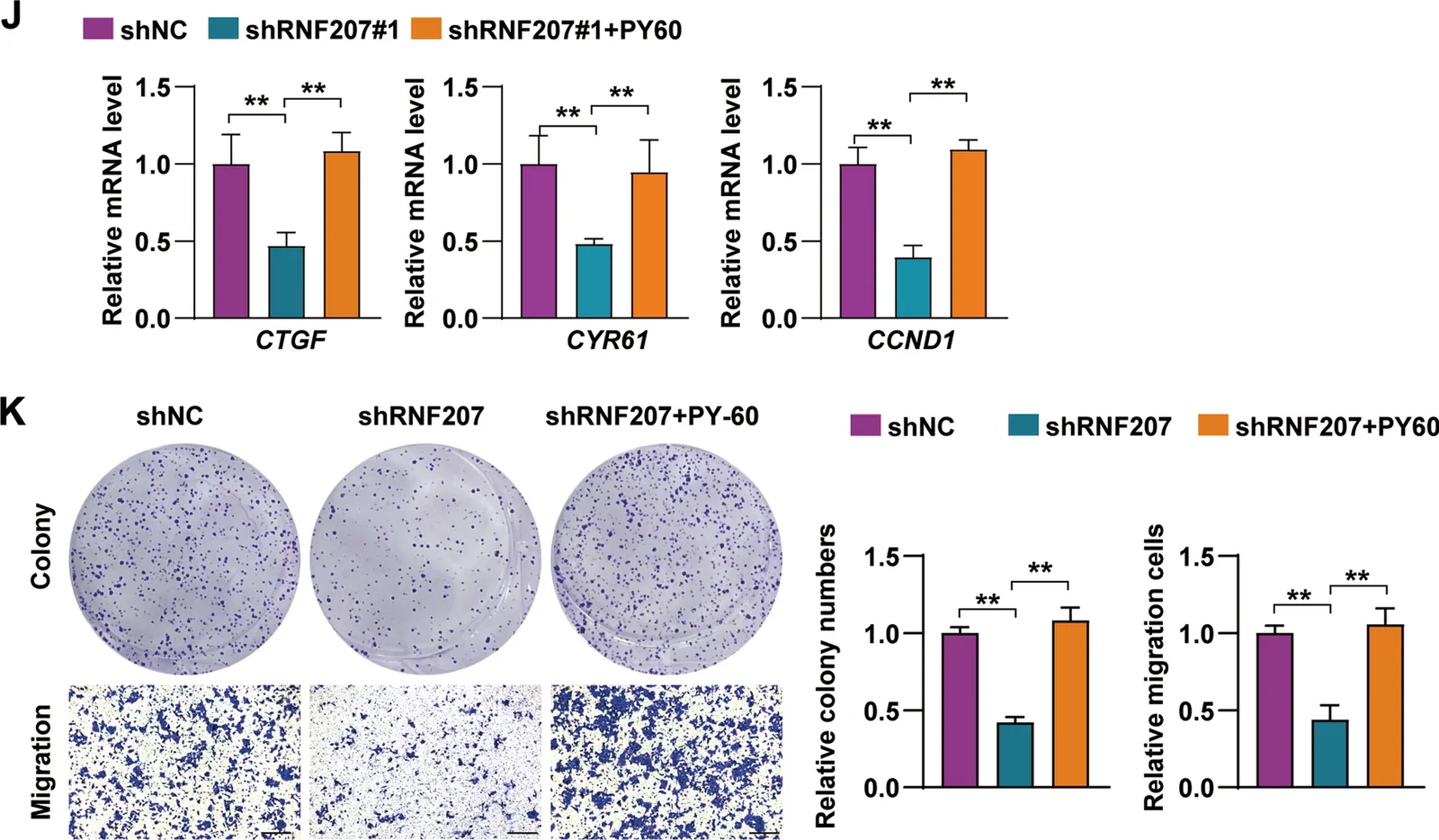
RNF207 promoted CRC cell progression via activating YAP transcriptional activity. **A–B** Western blot analysis of YAP, p-YAP, and TAZ proteins (left) and quantification results (right) in RNF207 overexpression (**A**) and knockdown (**B**) HCT116 cells. Actin served as a control. **C** Western blot analysis of p-YAP and RNF207 proteins (Up) and quantification results (down) in human colorectal cancer. Actin served as a loading control. **D** Representative immunohistochemical images of p-YAP in cancer tissues and adjacent tissues of colorectal cancer patients. Bar, 50 μm. **E** Western blot analysis of YAP and p-YAP (Ser127) protein levels in nuclear (left) and cytoplasmic fractions (right) of CRC cells with RNF207 overexpression (RNF207) or knockdown (shRNF207#1, shRNF207#2) compared to respective controls (Control, shNC). Lamin B and β-actin were used as loading controls for nuclear and cytoplasmic fractions, respectively. **F** Immunofluorescence staining of YAP (green) and Flag or RNF207 (red) in CRC cells with RNF207 overexpression or knockdown. Nuclei were stained with DAPI (blue). Bar, 10 μm. **G** Correlation analysis between YAP target genes (such as *CTGF, CYR61, CCND1, and BCL-2*) and RNF207 mRNA expression levels in TCGA colorectal cancer dataset. **H** qPCR analysis of YAP targets genes *(CTGF, CYR61, and CCND1)* expressions in RNF207 overexpression (left) and knockdown (right) HCT116 cells. The mRNA expression levels were normalized to *ACTB*. **I** qPCR analysis of YAP targets genes *(CTGF, CYR61, and CCND1)* expressions in cancer tissues and adjacent tissues of colorectal cancer patients. The mRNA expression levels were normalized to *ACTB*. **J** qPCR analysis of YAP targets genes *(CTGF, CYR61, and CCND1)* expressions in RNF207 knockdown HCT116 cells with or without PY-60. The mRNA expression levels were normalized to *ACTB* levels. **K** Representative images of colony formation assay, Transwell assay, and quantification results in RNF207 knockdown HCT116 cells with or without PY-60. Scale bar, 100 μm. For **A**, **C**, **H**, and I, two-tailed unpaired Student’s t-test was used. For **B, H, J**, and **K**, one-way ANOVA test was used. **P < 0.01; **P < 0.05; n.s., not significant

Numerous studies have shown that Hippo–YAP signaling pathway plays a key regulatory role in the occurrence and development of tumors, including colorectal cancer. In particular, YAP and TAZ are frequently activated in tumor cells to promote tumor promotion [10, 21]. We therefore examined the effect of RNF207 on Hippo–YAP signaling pathway, Western blot analysis results showed that the total protein levels of YAP and TAZ were not altered by RNF207 expression, but the phosphorylation level of YAP was significantly inhibited by overexpression of RNF207 while promoted by knockdown of RNF207 (Fig. 4A, B). Contrary to the elevated level of RNF207 protein observed in human colorectal cancer samples, the phosphorylation level of YAP was significantly decreased (Fig. 4C, D). Moreover, both nuclear-cytoplasmic fractionation and immunofluorescence staining demonstrated that RNF207 overexpression substantially enhanced YAP nuclear import, whereas RNF207 knockdown suppressed YAP nuclear translocation (Fig. 4E, F). It implied that RNF207 might exert its effects by modulating YAP transcriptional activity in colorectal cancer. To test this hypothesis, we initially analyzed the correlation between RNF207 expression and YAP downstream target genes using the TCGA COAD dataset. The results showed that RNF207 expression was positively correlated with CTGF, CYR61, CCND1, and BCL-2 (Fig. 4G). Consistently, YAP target genes such as *CTGF, CYR61, and CCND1* were significantly upregulated by RNF207 overexpression and downregulated by RNF207 knockdown (Fig. 4H). Additionally, these target genes were also upregulated in human CRC samples (Fig. 4I). Collectively, these results demonstrated that RNF207 affect YAP transcriptional activity.

To further investigate whether the effects of RNF207 on CRC cell proliferation and migration are dependent on the transcriptional activity of YAP, PY60, a specific activator of YAP transcriptional activity, was used in RNF207 knockdown HCT116 cells. qPCR analysis showed the reduced expressions of *MYC*, *AREG,* and *CTGF* genes by RNF207 knockdown were elevated after PY-60 treatment (Fig. 4J). Moreover, colony formation and Transwell assays respectively showed the inhibited cell proliferation and migration by RNF207 knockdown were increased by PY-60 (Fig. 4K). All these proved that RNF207 promoted CRC cell progression through activating YAP transcriptional activity.

### MST1 mediated the effect of RNF207 on the proliferation and migration of CRC cells

**Fig. 5:**
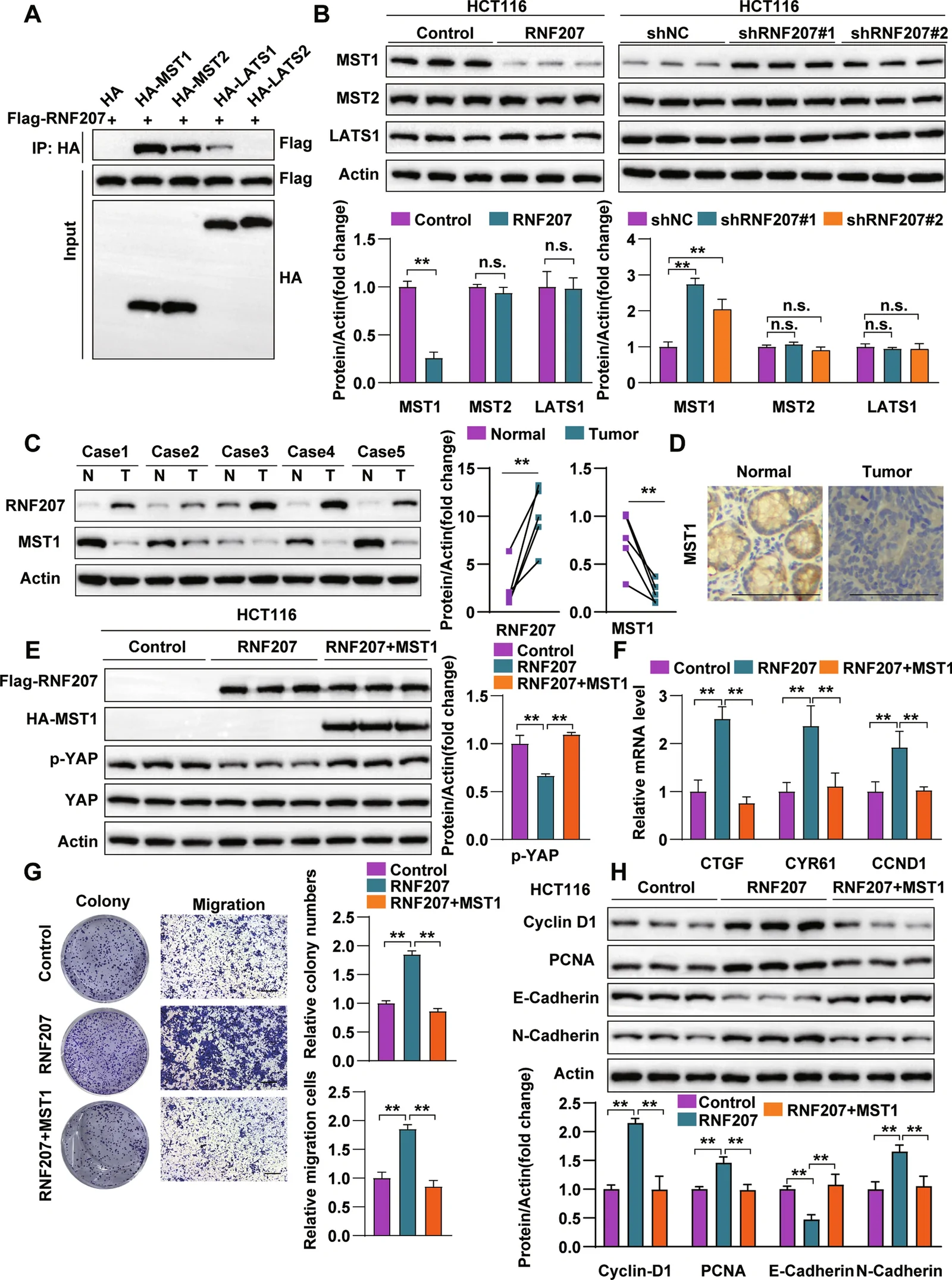
MST1 mediated the effect of RNF207 on the proliferation and migration of CRC cells.The result of Co-IP assays between RNF207 and MST1, MST2, LATS1, and LATS2 in HEK 293T cells. **B** The protein level and quantification results of MST1, MST2, and LATS1 in RNF207 overexpression and knockdown HCT116 cells. Actin served as a loading control. **C** The protein level and quantification results of MST1 and RNF207 in human colorectal cancer. Actin served as a loading control. **D** Representative immunohistochemical images of MST1 in cancer tissues and adjacent tissues of colorectal cancer patients. Bar, 50 μm. **E** Western blotting (left) and quantification (right) analysis of YAP proteins in RNF207 overexpression cell line with or without MST1. **F** qPCR analysis of *CTGF, CYR61 and CCND1* in RNF207 overexpression cell lines with or without MST1. The mRNA expression levels were normalized to *ACTB* levels. **G** Representative images of colony formation assay and Transwell assay (left) and quantification result (right) of RNF207 overexpression cell line with or without MST1. **H** Western blotting (left) and quantification (right) analysis of Cyclin-D1, PCNA, E-cadherin, and N-cadherin in RNF207 overexpression cell line with or without MST1. Actin served as a loading control. For **B** and **C**, two-tailed unpaired Student’s t-test was used. For **B, E, F, G**, and **H**, one-way ANOVA test was used. **P < 0.01; **P < 0.05; n.s., not significant

Since RNF207 is an E3 ubiquitin ligase and generally cannot phosphorylate its substrate directly, we next explored the relationship between RNF207 and the classical upstream regulators of YAP. Combining Co-IP results, we found that, except for LATS2, the MST1, MST2, and LATS1, all interact with RNF207 (Fig. 5A).Western blot results further revealed that only the protein level of MST1 was significantly altered upon differential expression of RNF207 (Fig. 5B). Notably, in contrast to the elevated RNF207 protein levels in CRC samples, MST1 protein levels were significantly decreased (Fig. 5C). Consistently, immunohistochemical results also revealed downregulation of both MST1 and p-YAP in human CRC samples (Fig. 5D). These results suggest that the MST1 may be mediate the regulation of p-YAP by RNF207.

To investigate whether MST1 mediated RNF207’s function, we conducted rescue experiment where MST1 and RNF207 were both overexpressed. We first examined the expression of YAP proteins, and found neither RNF207 nor MST1 altered the total YAP protein level, yet the decreased effect of RNF207 on phosphorylated YAP was reversed upon MST1 overexpression (Fig. 5E). Similarly, the increased abundances of *MYC*, *AREG,* and *CTGF* were reduced due to MST1 overexpression (Fig. 5F). Next, colony formation and Transwell assays showed the lager number of colonized and migrating cells due to RNF207 overexpression was obviously declined when MST1 was simultaneously overexpressed (Fig. 5G). Lastly, Western blotting results further showed the increased Cyclin-D1, PCNA, and N-cadherin by RNF207 were inhibited upon MST1 overexpression, while the decreased E-cadherin by RNF207 was elevated upon MST1 overexpression, facilitating the above conclusion (Fig. 5H). Taken together, we concluded that RNF207 exerted oncogenic roles in CRC relying on lowering MST1-mediated YAP activation.

### RNF207 directly interacted with MST1 and promoted MST1 degradation by K48-linked ubiquitination

**Fig. 6:**
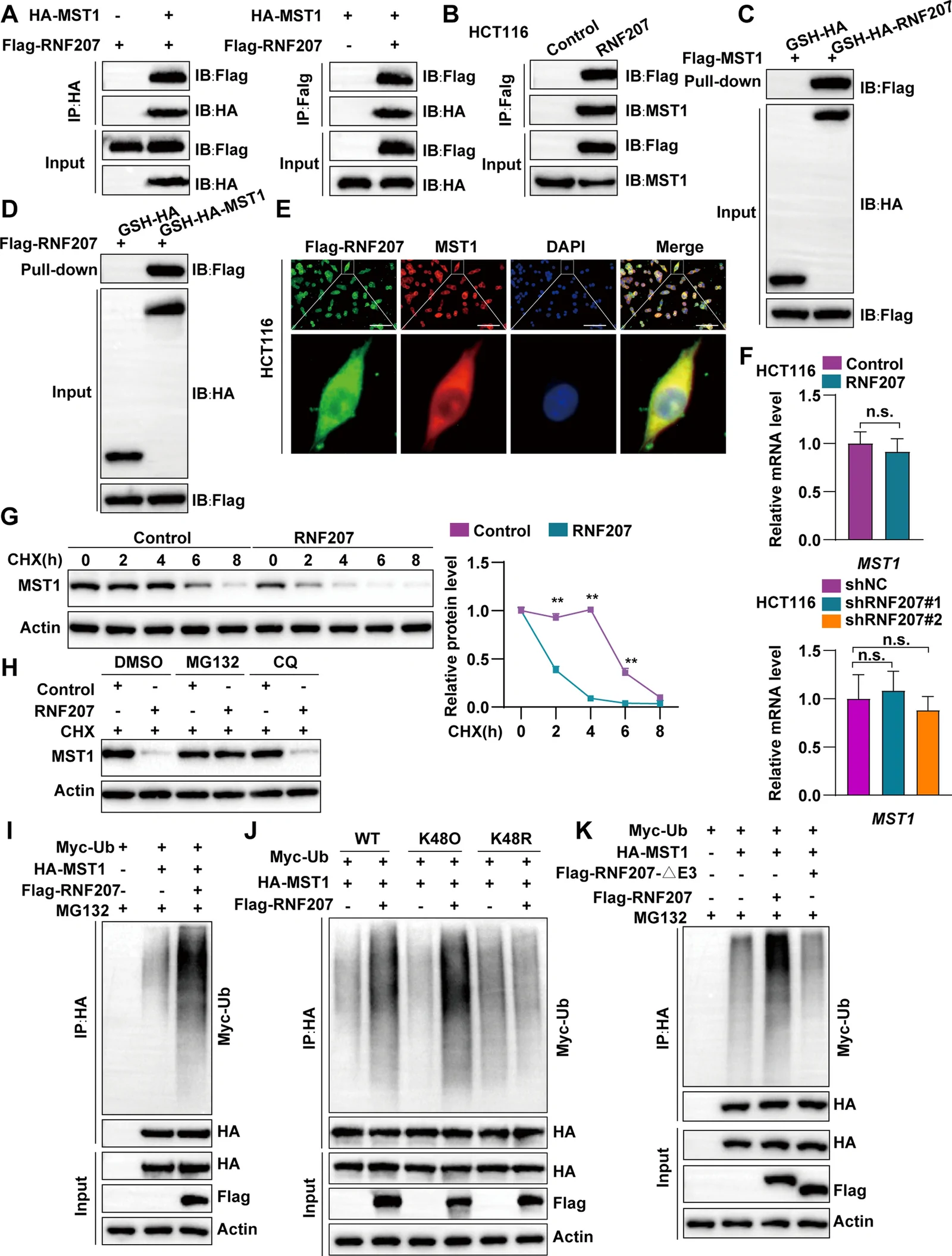
RNF207 directly interacted with MST1 and promoted MST1 degradation by K48-linked ubiquitination. **A** The result of Co-IP assays between RNF207 and MST1 in HEK293T cells. **B** The result of Co-IP assays between RNF207 and MST1 in HCT116 cells. **C–D** The result of GST-pulldown assay between RNF207 and MST1 in HEK293T cells. **E** Representative picture of RNF207 co-localization with MST1 in HCT116 cells. DAPI was used to indicate the nucleus and labeled in blue, MST1 in red, Flag-RNF207 in green. Scale bar, 100 μm. **F** The mRNA level of MST1 in RNF207 overexpression, RNF207 knockdown, and control HCT116 cells. The mRNA expression levels were normalized to *ACTB* levels. **G** The protein level of MST1 in RNF207 overexpression and control HCT116 cells with time-gradient addition of CHX(50 μmol). Actin served as a loading control. **H** The protein level of MST1 in RNF207 overexpression and control HCT116 cells treated with CHX and MG132 or CQ. Actin served as a loading control. **I** Results of ubiquitination of MST1 by RNF207 in HEK293T cells transfected with RNF207, MST1, and ub expression plasmids, respectively. **J** Results of ubiquitination of MST1 by RNF207 in HEK293T cells transfected with RNF207, MST1, and K48-linked ub mutant expression plasmids, respectively. **K** Results of ubiquitination of MST1 by RNF207 in HEK293T cells transfected with RNF207, RNF207 variant deleting E3 ligase function domain, MST1, and ub expression plasmids, respectively. For F and G, two-tailed unpaired Student’s t-test was used. **P < 0.01; **P < 0.05; n.s., not significant

To explore how RNF207 regulated MST1, we first investigated the interaction between RNF207 and MST1. The result of Co-IP assay proved that RNF207 interacted with MST1 in HEK293T cells and HCT116 cells (Fig. 6A–B). Moreover, GST-pulldown assays proved MST1 and RNF207 physically interacted (Fig. 6C–D). The immunofluorescent staining result showed that MST1 and RNF207 colocalized in cytoplasm of HCT116 cells, satisfying the space requirement of their interaction (Fig. 6E). All these proved RNF207 could interact with MST1in CRC cells and reduced the protein level of MST1. Then, we examined the mRNA level of MST1 in different CRC cells. We found the transcriptional level of MST1 was not changed by RNF207 overexpression (Fig. 6F). This indicates that RNF207 regulates MST1 through post-translational modification. To investigate the mechanism of RNF207 regulating MST1, we supplemented cycloheximide (CHX), the inhibitor of protein synthesis, into RNF207 overexpressing and its control cell lines and found RNF207 shortened the half-life of MST1 protein, indicating RNF207 decreased MST1’s protein level by promoting its degradation (Fig. 6G). To distinguish which degradation pathway RNF207 relied on, we adopted the proteasome inhibitor MG132 and the lysosomal pathway inhibitor chloroquine (CQ) in RNF207 overexpressing and its control cell lines together with CHX and found MG132 but not CQ inhibited the degradation of MST1 by RNF207, proving RNF207 promoted MST1’s degradation through ubiquitin–proteasome pathway (Fig. 6H). Further, ubiquitination assay clearly showed the ubiquitination of MST1 was much enhanced by RNF207 (Fig. 6I). Applying mutated forms of ubiquitin, we found RNF207 failed to link K48R (Ub only Lys48 residue was mutated) to MST1, but could link wild-type Ub and K48O (Ub with the intact Lys48 residue alone) to MST1, indicating RNF207 predominantly promoted K48-linked ubiquitination of MST1 (Fig. 6J). Utilizing truncated RNF207 variant lacking E3 ligase domain, we demonstrated that deletion of this domain abolished RNF207-mediated ubiquitination of MST1 (Fig. 6K). Taking all into account, we concluded RNF207 promoted MST1 degradation by K48-linked ubiquitination dependent on its ubiquitin activity.

### Reducing the expression of RNF207 significantly inhibits the growth of CRC in vivo

**Fig. 7:**
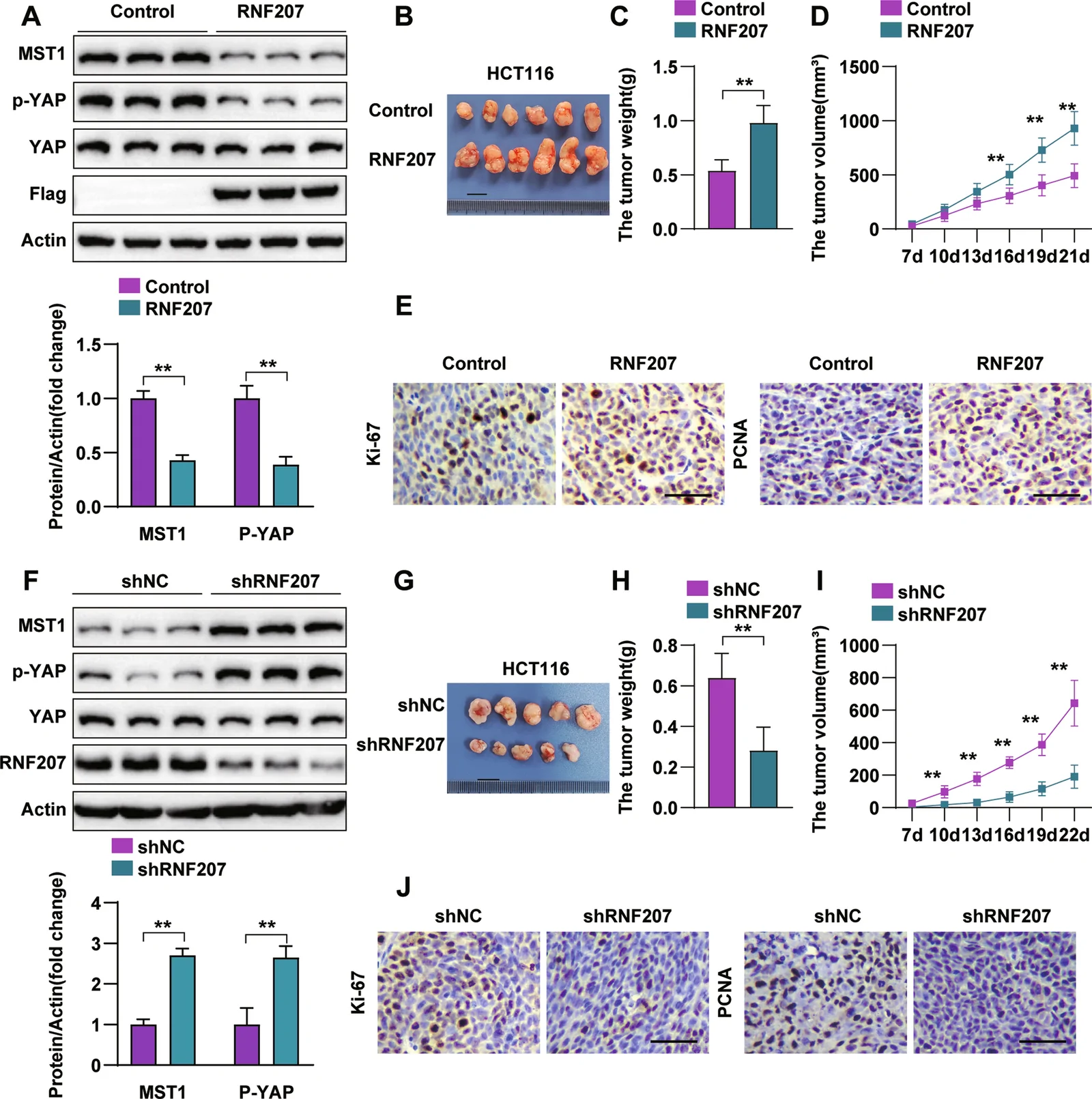
RNF207 facilitated the CRC *growth *in vivo*.* **A** Western blotting analysis of MST1, p-YAP, YAP, and Flag protein levels in xenograft tumors derived from RNF207 overexpression HCT116 cells and controls. **B** Representative images of subcutaneous xenograft tumors derived from RNF207 overexpression HCT116 cells and controls. Bar, 1 cm. **C** The final tumor weight and **D** the volume of the tumors derived from RNF207 overexpression HCT116 cells and controls. **E** Representative immunohistochemical staining images of Ki67 and PCNA in xenograft tumors derived from RNF207 overexpression HCT116 cells and controls. Bar, 50 μm. **F** Western blotting analysis of MST1, p-YAP, YAP, and RNF207 expressions in xenograft tumors derived from RNF207 knockdown HCT116 cells and controls. **G** Representative images of subcutaneous xenograft tumors derived from RNF207 knockdown HCT116 cells and controls. Bar, 1 cm. **H** The final tumor weight and **I** volume of the tumors derived from RNF207 knockdown HCT116 cells and controls at different time points. **J** Representative immunohistochemical staining images Ki67 and PCNA in xenograft tumors derived from RNF207 knockdown HCT116 cells and controls. Scale bar, 50 μm. For **C**,** E**,** H**, and **J**, two-tailed unpaired Student’s t-test was used. **P < 0.01; **P < 0.05; n.s., not significant

To validate the effect of RNF207 on the growth of colorectal cancer, we further established subcutaneous xenograft tumor model in nude mice. The Western blot results showed that in the tumor tissues with overexpression of RNF207, the protein level of MST1 and the phosphorylation level of YAP were significantly reduced (Fig. 7A). The tumors of RNF207 overexpressing mice were significantly larger than those of control mice, both in terms of tumor volume and tumor weight (Fig. 7B–D). What is more, the results of histopathological staining showed that the positive cells of Ki-67 and PCNA in the tumor tissues of RNF207 overexpression mice were significantly more than those of the control group (Fig. 7E). On the other side, HCT116 cells harboring RNF207 knockdown and control vectors were also subjected to subcutaneous xenograft tumor model. The Western blot results showed that in the tumor tissues with knockdown of RNF207, the protein level of MST1 and the phosphorylation level of YAP were significantly increased. (Fig. 7F). More importantly, RNF207 knockdown mice had significantly smaller tumor volume and weight than control mice (Fig. 7G–I). The immunohistochemical staining also showed that the positive cells of Ki67 and PCNA in RNF207 knockdown group were much fewer than control group (Fig. 7J). These data indicate that inhibiting RNF207 can alleviate the growth of colorectal cancer in vivo.

## Discussion

Colorectal cancer (CRC) represents one of the most prevalent tumors worldwide. Although progress in research has translated into reduced mortality of CRC, the prediction of early onset of CRC and deep understanding of genetic and pathologic alterations in CRC are still the hotspot of researches [22]. In the present research, we found *RNF207* was upregulated in CRC cancerous tissues. Furthermore, we identified that the upregulation of RNF207 is driven by FOXM1, a well-documented oncogenic transcription factor in CRC that plays pivotal roles in promoting tumor growth, metastasis, and progression [20]. Lower expression of *RNF207* was correlated to good prognosis of CRC patients, highlighting the potential role of RNF207 as a biomarker for CRC prognosis. Moreover, we comprehensively investigated and proved RNF207 exerted oncogenic function in CRC through activating the transcriptional activity of YAP, deepening the understanding of molecular mechanism in CRC progression. Our findings prospect new therapeutic strategy development for CRC treatment.

RING finger (RNF) proteins are one group of E3 ubiquitin ligase and involved in the pathogenesis of various diseases including cancer [23]. Concerning CRC, several RNF proteins are identified and proved to be oncogenic. For example, RNF6 is upregulated in 73.5% (147/200) of patients with colorectal cancer and promotes cell growth and EMT through binding and ubiquitination of transducin-like enhancer of split 3 (TLE3), which is transcriptional repressor of the β-catenin/TCF4 complex in Wnt/β-catenin pathway [24]. Besides, RNF183 and RNF220 are also found highly expressed in tumor tissues and facilitate growth, migration, and invasion of CRC cells [25, 26]. Consistent with these researches, RNF207 is also found highly expressed in CRC and promotes cell proliferation and metastasis.

The Hippo–YAP pathway is crucial for the initiation of tumor formation. As the core downstream transcription factor of this pathway, YAP’sphosphorylation inhibits its nuclear translocation and thereby inhibits its transcriptional activity [10]. In our study, although the TCGA data analysis revealed that the correlation between RNF207 and the Hippo–YAP pathway was not significant (not shown), RNF207 was positively correlated with the downstream target genes of YAP. And it was found that RNF207 inhibited the phosphorylation of YAP, promoting its nuclear translocation and transcriptional activity. Further research revealed that RNF207 promoted the phosphorylation of YAP by ubiquitination and degradation of its upstream kinase MST1. This reflects that RNF207 specifically regulates the activity of YAP through post-translational degradation of MST1 without widely affecting the transcriptional expression of other Hippo components.

Critical for the initiation of tumorigenesis, the fundamental constituents in Hippo pathway, including MST1 and YAP, frequently experience genetic mutations and modified expressions in CRC [27]. Previously, MST1 was found downregulated in CRC, and overexpression of MST1 induced CRC apoptosis and impaired cell proliferation and migration through inhibiting Bnip3-related mitophagy via activating JNK/p53 pathway [28]. Genetic deletion of YAP promoted the growth of organoids, patient-derived xenografts, and mouse models of primary and metastatic CRC, proving the role of YAP as a tumor suppressor in the adult colon [29]. MST1/YAP axis was also proved to account for the underlying mechanism of metallothionein 2A (MT2A) and LOX-like 1 (LOXL1) in CRC, where MST1 was phosphorylated and activated to phosphorylate YAP, thereby preventing CRC progression [30, 31]. In the present study, we identified MST1/YAP axis underlay the function of RNF207 in CRC, because overexpression of MST1 in the background of RNF207 overexpression relieved its oncogenic effects on cell proliferation and migration, further underlining Hippo pathway’s pivotal roles in CRC.

As core component of Hippo pathway, MST1 undergoes multiple post-translational modifications, including phosphorylation, acetylation, and ubiquitination. In oxidative stress-induced neurons, tyrosine kinase c-Abl phosphorylates MST1 at Y433, triggering stabilization and activation of MST1 and finally promoting cell death [32]. MST1 can also autophosphorylate itself at T183, which is enhanced by homodimerization [33]. Moreover, MST1 could be acetylated on its lysine 35 residue in cells, and deacetylation of MST1 mediated by HBXIP (hepatitis B X-interacting protein)-enhanced HDAC6 (histone deacetylase 6) results in MST1 degradation via chaperone-mediated autophagy (CMA) promoting breast cancer growth [34]. SIRT7 (NAD-dependent protein deacylase sirtuin-7) directly binds to and deacetylates MST1, priming acetylation-dependent MST1 ubiquitination and protein degradation in hepatocellular carcinoma [35]. Besides, TRIM21 interacted and induced ubiquitination of MST1 in hepatocellular carcinoma [36]. E3 ubiquitin ligase Cullin 3 can also promote the ubiquitination and degradation of MST1 without changing the expression of phosphorylated MST1 in oral squamous cell carcinoma [37]. Consistently, here we found RNF207 ubiquitinated MST1 and promoted its degradation in colorectal cancer cells, broadening our understanding of MST1’s post-translational modifications and highlighting the critical roles of MST1 protein stability. Further, utilizing mutated types of ubiquitin and truncated RNF207 variant, we identified RNF207 promoted K48-linked ubiquitination of MST1 relying on E3 ligase domain, describing a more detailed ubiquitination process.

Several Hippo signaling-related agonists and inhibitors show great potential for therapeutic interventions, especially drugs targeting TEAD advancing to clinical trials. For example, central pocket TEAD inhibitors such as IK-930 and VT3989, which target solid tumors like lung, pancreatic, and colorectal cancers, as well as NF2-enriched malignant mesothelioma (NCT05228015 and NCT04665206) are underway in clinical trials [38]. As for agonists of MST1, EMT inhibitor-1, also known as C19, directly activates MST1 and AMPK, demonstrating potential in specifically suppressing tumor growth in certain contexts [39]. More researches are focusing on the inhibitors of MST1, facing the challenges in achieving high selectivity for MST1/2 to minimize off-target effects [40]. Our findings reveals RNF207 degradation of MST1 to promote CRC cell progression, and elevating MST1 activity might benefit the treatment of CRC, highlighting the manipulation of RNF207–MST1 axis prospects new therapeutic strategy development.

Despite the consistent evidence from both in vitro and in vivo experiments supporting the oncogenic role of RNF207 in colorectal cancer, validation using patient‑derived organoids or murine colorectal cancer organoids would further strengthen the pathophysiological relevance of our findings. While we fully concur that organoid models offer a more physiologically relevant platform for studying tumor biology and therapeutic response, establishing such a system requires specialized expertise, infrastructure, and optimized culture conditions that are currently unavailable in our laboratory. Therefore, organoid‑based validation was not performed in this study. We consider this as a limitation, and in our future work, we will endeavor to establish the organoid platform to complement and extend the current conclusions.

## Conclusion

RNF207 is upregulated in colorectal cancer tissues, and its upregulation is associated with poor prognosis. Preclinical studies showed that RNF207 significantly promoted the proliferation and migration of colorectal cancer cells by inhibiting the phosphorylation of YAP through K48-linked deubiquitination and degradation of MST1. Targeting RNF207–MST1–YAP axis may represent a potential therapeutic for colorectal cancer treatment. Our research has revealed new functions and mechanisms of RNF207 in colorectal cancer, and has provided potential targets for the prevention and treatment of CRC.

## Supplementary Information

Below is the link to the electronic supplementary material.

Supplementary file1 (DOCX 18 KB)

## Abbreviations

AREG: Amphiregulin
CCK8: Cell counting kit 8
CRC: Colorectal cancer
CTGF: Extracellular-signal regulated kinases
CHX: Cycloheximide
EMT: Epithelial–mesenchymal transition
MST1: Mitogen-activated protein kinases
MYC: MYC proto-oncogene
RNF207: RING finger protein 207
TAZ: Transforming growth factor-β-activated kinase 1
TCGA: The cancer genome atlas
YAP: Yes1 associated transcriptional regulator
