# Supplementary material for: RNF207 promotes colorectal cancer growth by regulating the Hippo–YAP pathway via enhanced MST1 ubiquitination and degradation
Source: J Gastroenterol. 2026 May 27;61(10):1431–49. doi: 10.1007/s00535-026-02440-2 (PMC13615925; doi:10.1007/s00535-026-02440-2)
Supplement: Supplementary file 1 [file 535_2026_2440_MOESM1_ESM.docx]

[SupplementaryTable S1](https://oup.silverchair-cdn.com/oup/backfile/Content_public/Journal/cardiovascres/119/1/10.1093_cvr_cvac039/1/cvac039_supplementary_data.zip?Expires=1747884852&Signature=MDSJ~MX8hVqvXj~1q~1wM0KlVEqsSZxjwWCqM63VRNoZucyT6VD9pCVfgxe1EUmJemabD49Ij1gtGC1A~EPshCmnqPZts~Wz1cZi0igQPlOPoqgiqderkIivVlwH7nR1GpNYKVyZgIK8-3z4SsWPohRPFXHuswe2RWWKYTlF5T6OAcISW-mZcDm2aBnGtTBE8jxv6mIx8Rj4mX4MXfaEwblA8s5ARbw4CeXCxBli4MAEDI299fkwuT-GkgGXUX0uvo6PuOXj31n~o743~lffOAgfzAr-VUdkZwmOk4iA1dEsk1oA6vcaXkUWZX8-M6FcfeoSqgHvkyURNG~ZjHA1rg__&Key-Pair-Id=APKAIE5G5CRDK6RD3PGA). The q-PCR primers Sequences

| Gene name | Sequences (5’-3’) | |
| --- | --- | --- |
| *RNF207* (human) | Forward | CGCAGAGCCCTACCTCTTG |
|  | Reverse | CACGTAAGCCGATTCCAGGTC |
| CCND1 (human) | Forward | CAGATCATCCGCAAACACGC |
|  | Reverse | AGGCGGTAGTAGGACAGGAA |
| *CYR61* (human) | Forward | GAAGCGGCTCCCTGTTTTTGG |
|  | Reverse | CTTGCTGCATTTCTTGCCCTT |
| *CTGF* (human) | Forward | CCGGGTTACCAATGACAACG |
|  | Reverse | TTGGGAGTACGGATGCACTT |
| *MST1* (human) | Forward | GGAAGTGTGGGAGGGTCTG |
|  | Reverse | TATACGCTGCCATAGGACCCTT |
| *ACTB* (human) | Forward | CCGGGACCTGACTGACTACC |
|  | Reverse | AGGAAGGCTGGAAGAGTGC |
| *FOXM1* (human) | Forward | ATACGTGGATTGAGGACCACT |
|  | Reverse | TCCAATGTCAAGTAGCGGTTG |

[Supplementary](https://oup.silverchair-cdn.com/oup/backfile/Content_public/Journal/cardiovascres/119/1/10.1093_cvr_cvac039/1/cvac039_supplementary_data.zip?Expires=1747884852&Signature=MDSJ~MX8hVqvXj~1q~1wM0KlVEqsSZxjwWCqM63VRNoZucyT6VD9pCVfgxe1EUmJemabD49Ij1gtGC1A~EPshCmnqPZts~Wz1cZi0igQPlOPoqgiqderkIivVlwH7nR1GpNYKVyZgIK8-3z4SsWPohRPFXHuswe2RWWKYTlF5T6OAcISW-mZcDm2aBnGtTBE8jxv6mIx8Rj4mX4MXfaEwblA8s5ARbw4CeXCxBli4MAEDI299fkwuT-GkgGXUX0uvo6PuOXj31n~o743~lffOAgfzAr-VUdkZwmOk4iA1dEsk1oA6vcaXkUWZX8-M6FcfeoSqgHvkyURNG~ZjHA1rg__&Key-Pair-Id=APKAIE5G5CRDK6RD3PGA) [Table S2](https://oup.silverchair-cdn.com/oup/backfile/Content_public/Journal/cardiovascres/119/1/10.1093_cvr_cvac039/1/cvac039_supplementary_data.zip?Expires=1747884852&Signature=MDSJ~MX8hVqvXj~1q~1wM0KlVEqsSZxjwWCqM63VRNoZucyT6VD9pCVfgxe1EUmJemabD49Ij1gtGC1A~EPshCmnqPZts~Wz1cZi0igQPlOPoqgiqderkIivVlwH7nR1GpNYKVyZgIK8-3z4SsWPohRPFXHuswe2RWWKYTlF5T6OAcISW-mZcDm2aBnGtTBE8jxv6mIx8Rj4mX4MXfaEwblA8s5ARbw4CeXCxBli4MAEDI299fkwuT-GkgGXUX0uvo6PuOXj31n~o743~lffOAgfzAr-VUdkZwmOk4iA1dEsk1oA6vcaXkUWZX8-M6FcfeoSqgHvkyURNG~ZjHA1rg__&Key-Pair-Id=APKAIE5G5CRDK6RD3PGA). The antibodies

| Antibody | Catalogue number | Manufacturer | Source | Dilution |
| --- | --- | --- | --- | --- |
| RNF207 | HPA042535 | Sigma Aldrich | rabbit | 1:1000 |
| Cyclin-D1 | A19038 | Abclonal | rabbit | 1:1000 |
| PCNA | A12427 | Abclonal | rabbit | 1:5000 |
| E-cadherin | A11492 | Abclonal | rabbit | 1:1000 |
| N-cadherin | A19083 | Abclonal | rabbit | 1:1000 |
| Ki67 | sc-7846 | santa cruz | Goat | 1:200 |
| YAP | A19134 | Abclonal | rabbit | 1:1000 |
| p-YAP | ET1611-69 | HuaBio | Rabbit | 1:5000 |
| TAZ | A23034 | Abclonal | rabbit | 1:1000 |
| MST1 | A21842 | Abclonal | rabbit | 1:1000 |
| p-MST1 | AP1094 | Abclonal | rabbit | 1:1000 |
| Myc | M047-3 | MBL | mouse | 1:1000 |
| HA | AE105 | Abclonal | rabbit | 1:2000 |
| Flag | M185-3 | MBL | mouse | 1:1000 |
| Actin | AC026 | Abclonal | rabbit | 1:10000 |
| LAMINB | A1910 | Abclonal | rabbit | 1:1000 |
